# Understanding the etiology of ventral body wall defects

**DOI:** 10.1186/1753-6561-6-S4-O10

**Published:** 2012-07-09

**Authors:** H Ghorab, J Thompson

**Affiliations:** 1 Royal College of Surgeons in Ireland-Bahrain; 2 University College Dublin, Belfield, Dublin 4, Ireland

## Introduction

Ventral body wall (VBW) defects are observed in 1 in 3000 births. The main types of defects include body wall dysplasia, gastroschisis, omphalocele and primary thoracoabdominoschisis. There is little evidence suggesting that genetic changes are associated with these defects. An important number of these occur as a result of exposure to chemical pollutants or radiation. In this project chick embryos exposed to cadmium were used as a model of VBW defect. The project aimed to identify when the ventral wall in chicks is fully closed and to investigate the migration of different cells and tissues in normal control embryos and compare it to embryos exposed to cadmium.

## Methods

Fertile eggs were collected from a local hatchery. The embryos were placed in an incubator set at 37 °C, cadmium was administered ex ovo. The embryos were then fixed in 4F1G solution for at least 24 hours, following, the embryos were processed in paraffin wax which is firm enough to support subsequent cutting. The blocks were sectioned and attached to slides using a microtome. Finally the slides were stained using hematoxylin and eosin stains, hematoxylin stains the nuclei in a blue/black color whereas eosin stains cell cytoplasm and most connective tissue fibers in shades of pink, orange and red.

## Results

Gross anatomical differences between the controls and cadmium treated embryos were present, such as protrusion of abdominal contents out of the abdomen as well as abnormal body axial development. Microscopic differences include a large notochord, an underdeveloped dermomyotome and a disorganized sclerotomal migration.

## Conclusions

VBW defects are uncommon however if present they may lead to an increase in neonatal morbidity and less frequently mortality. We have shown through this project that cadmium induced gross and microscopic anatomical defects in chick embryos. These findings imply that vertebral malformation may occur due to abnormal development of the somites giving rise to body axial deformity, which may directly contribute to primary VBW defects. Additional research is warranted to demonstrate pathways, which may be involved in normal formation of the ventral body wall.

